# A Review on Medicinal and Ethnomedicinal Uses, Biological Features, and Phytochemical Constituents of *Sesbania sesban* L. Merr., A Nitrogen-Fixing Plant Native to the Republic of Chad

**DOI:** 10.1155/2024/1225999

**Published:** 2024-01-17

**Authors:** Ousman Brahim Mahamat, Saoud Younes, Brahim Boy Otchom, Steven Franzel, Al-Djazouli Ouchar Mahamat Hidjazi, El ismaili Soumaya

**Affiliations:** ^1^Laboratory of Biology, Ecology and Health, Faculty of Sciences, Abdelmalek Essaâdi University, Tetouan, Morocco; ^2^National Federation Associations of Healers and Practitioners of Medicine, The Ministry of Public Health of Chad, N'Djamena, Chad; ^3^Faculty of Human Health Sciences (FSSH), University of N'Djamena, N'Djamena, Chad; ^4^Toumaï University of N'Djamena, N'Djamena, Chad; ^5^Independent Consultant, Maitland, FL, USA; ^6^Faculty of Exact Applied Sciences, Department of Earth Sciences, N'Djamena University, N'Djamena, Chad; ^7^Laboratory of Geology and Oceanology, Geology Department, Faculty of Sciences, Abdelmalek Essaâdi University, Tetouan, Morocco; ^8^Laboratory of Innovative Technologies, Civil Engineering Department, National School of Applied Sciences ENSA-Tangier, Abdelmalek Essaâdi University, Tetouan, Morocco

## Abstract

This study reports on a literature review of the leguminous tree *Sesbania sesban* (L.) Merr which is found in the N'djamena region, the Republic of Chad. The study focused on *S. sesban*'s medicinal and ethnomedicinal uses, biological features, and phytochemical constituents to assist in future evaluations. A literature review was conducted using academic websites, such as Science Direct and Springer, online international plant databases, and data from national herbaria. *S. sesban* is a perennial shrub or tree that measures 3-4 m in height. This species is becoming rare in N'djamena but can be found in the rainy season, while in winter, it occurs mainly in ponds (called the Chadian dialect “Bouta”) and on the shores of the Chari and Logone rivers. The local inhabitants in Chad use the species as medicine, livestock feed, and fuelwood and for improving soil fertility and repelling desert encroachment. Traditional healers use its leaves to treat breast cancer and edema. *S. sesban* is an essential species native to the Republic of Chad that needs conservation and valorization. Viewing its importance and rarity in N'djamena , a strategy for replanting the species in gardens, homes, and fields around N'djamena and other regions of Chad is recommended.

## 1. Introduction

The taxonomic diversity of legumes is enormous. In addition, they provide important benefits to humans including food, medicines, and environmental services. For example, leguminous food grains include beans (*Phaseolus vulgaris* L.), peas (*Pisum sativum* L.), soya beans (*Glycine max* L.), and forage legumes such as clover (*Trifolium repens* L.), sainfoin (*Onobrychis* Mill.), and *S. sesban* L. Merr. These plants (leguminosae) also fix atmospheric nitrogen via their symbiotic association with soil bacteria, belonging to the genera *Rhizobium*, *Azorhizobium*, *Bradyrhizobium*, and *Sinorhizobium* [1]. They can be used for soil improvement [2]. Nitrogen is commonly the most limiting element in food production and one of the most expensive in fertilizers. This special ability of leguminous crops to work symbiotically with rhizobia to produce nitrogen is becoming increasingly important in world agriculture [3].

Some forage legumes such as *S. sesban* are used in agroforestry systems in tropical regions including sub-Saharan Africa. They are also used for other purposes such as stakes, fuelwood, and reducing soil erosion. Land management practices featuring legumes include cereal-legume intercropping, relay cropping, biomass transfer, and fodder banks [3–6].

Leguminous species (including *S. sesban*) also play a great role in reforestation development programs in arid areas and the fight against desertification [2, 7, 8]. They are suitable alone or mixed with other species [9].

In Africa, many indigenous species such as *S. goetzei* Harms, *S. keniensis* J. B. Gillett, *S*. *rostrata* Bremek and Oberm, and *S. sesban* represent the genus *Sesbania.* These species, and particularly *S. sesban,* possess several desirable characteristics that make them suitable for use as multipurpose trees in farming systems [10]. *S. sesban* is considered suitable to alternate and/or intercrop with other agricultural species. It grows fast and efficiently recycles available nutrients within the system, thus shortening the time required to restore fertility [11, 12].

In Chad, the genera *Sesbania* are represented by many species such as *S. sesban*, *S. microphylla* Harms ex Phill and Hutch, *S. leptocarpa,* D.C., *S. pachycarpa,* D.C., *S. pubescens* D.C., *S. rostrata* Brem. and Oberm., *S. sesban* (L.) var. *nubica* Chiov., *S. sesban* subsp. *punctata* D.C. and Gillett, and *S. dalzielii* E. Phillips and Hutch [13, 14]. *Sesbania* species found in Chad include *S. sesban*, *S. dalzielii*, *S. rostrata*, *S. leptocarpa*, *S. sericea* (Willd.) (*S. pubescens* DC), *S. hepperi* J. B. Gillett, *S. cannabina* (Retz.) Pers., *S. tetraptera* Hochst. Ex-Baker, *S. pachycarpa* DC, and *S. microphylla* Harms [14].

The objective of this paper is to report medicinal and ethnomedicinal use, the biological activity, and the phytochemical constituents of *S. sesban* based on a literature review. Potential uses of the species in Chad and other countries are reported in this paper. This study contributes updated information concerning this useful but less exploited plant with the purpose of helping develop and conserve it in Chad.

## 2. Materials and Methods

The authors conducted a review of the literature on *S. sesban*, its applications, biochemistry, and interactions with other organisms, and its environment. Sources for the review included various academic websites such as Science Direct and Springer, online international plant databases, and data from herbaria in Chad such as the herbarium of the Institut Supérieur de Sciences de l'Education de N'N'djamena (ISSED) and the herbarium of Toumai University (N'N'djamena).

## 3. Results

### 3.1. Features of *S. sesban* and Name

**Figure 1:**
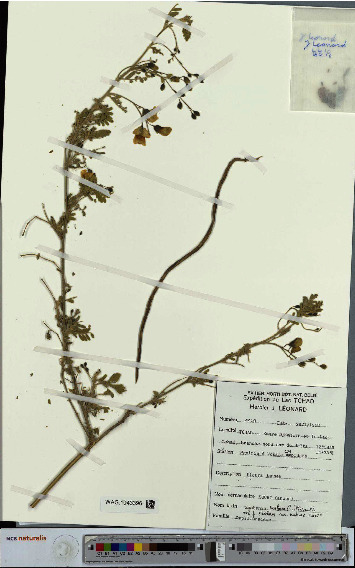
The botanical mission carried out on *S. sesban* in the Lake Chad area by Léonard in 1968 [19].

*Sesbania* is a Persian term, and in the Arabic language, it is Seysaban. The original taxon was created by Antonio Jose Cavanilles (1745–1804) and modified by George Bentham in 1859 in his book *Flora brasilensis* [15]. However, the names *Sesbania* [16], Seseban, and Sesban already existed [15]. Related names or synonyms are *S. Sesban* var. *nubica* Chiov [5, 17] or *S. aegyptiaca* auct [14, 18]. The local name in Chad is “Torotoro” (in Chadian Arabic) (Ousman B. M., 2024) (unpublished). It is also called “Surridj,” “Surridj-alkoubar,” and “Surridj-addougag” (Patrice 1997). Léonard in his botanical mission carried out on *S. sesban* in the Lake Chad area in 1968 reported the local name of “Souri” to S. sesban (Figure 1). Gaston and Fotuis [13] also reported other local names of *S. sesban* in different local Chadian dialects which are “prepre” (in Baguirmi dialect), “sinu” (in gabri kemdé dialect), “gegelek” (in Massa-moulouhi dialect) and “dao dao” or “doo” (in Sara doba dialect).  Table 1 shows the taxonomic classification of *S. sesban*.

#### 3.1.1. Distribution of *S. sesban* in Africa, Chad, and Other Countries

**Figure 2:**
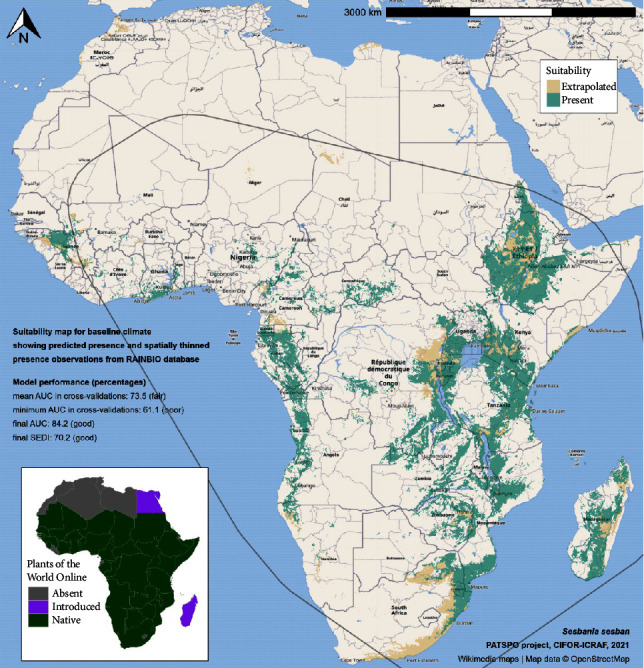
*S. sesban* Merr. (L.) is native to Chad and other countries [25].

The genus *Sesbania* (Fabaceae or Papilionaceae) consists of about 50 species of fast-growing trees and is composed of annual shrubs and perennial woody plants that are widespread in the tropics and subtropics [22], with a large number of accessions collected [10, 23]. Some 33 species are found in Africa, distributed between central and eastern Africa. *S. sesban* is widely distributed in semiarid to subhumid regions throughout the continent [18]. It develops in the wild in most geographical zones of Africa and in many different soil types [23]. Orwa et al. [6] reported that the other African countries where *S. sesban* is found in habitat are Chad, Egypt [14], Kenya, and Uganda. More recent databases on species distribution such as the Plants of the World Online [24] and Atlas World Agroforestry (Climate Change Atlas) [25] showed that the native range of this species *S. sesban* is Tropical and South Africa, Arabian Peninsula, and Indian Subcontinent. It is a shrub or tree that grows primarily in the seasonally dry tropical biome. Dufour-Dror [26] reported that *S. sesban* is an invasive species in Israel and the U.S. state of Hawaii [26]. It has become naturalized in many of the countries where it is cultivated and is characterized by very rapid early growth [27] (Figure 2).

**Figure 3:**
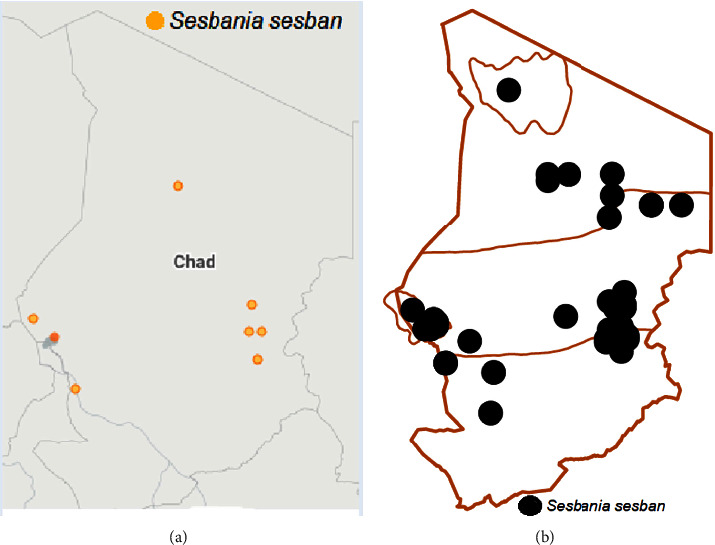
(a) In the orange color, zones of Chad where *S. sesban* is found [28]. (b) In the black color, zones of Chad where *S. sesban* is found according to [14].

In Chad, *Sesbania* species include *S. sesban* (Figure 3) and *S. microphylla* Harms ex. Phill and Hutch, *S. leptocarpa* D.C., *S. pachycarpa* D.C., *S. pubescens* D.C., *S. rostrata* Brem. and Oberm., *S. sesban* (L.) var. *nubica* Chiov., *S. sesban* subsp. *punctata* D.C. and Gillett, and *S. dalzielii* E. Phillips and Hutch [13, 14]. Chad is a centre of diversity for some of these species. However, *Sesbania* species have not been fully exploited as multipurpose plants in many central African countries [13]. As reported by César and Chatelain [14] and [21], *S. sesban* is found in different niches in Chad such as on riverbanks, in stream beds and wetlands, and around the water sources of Borkou and Ennedi (Figure 3).

#### 3.1.2. Habitat and Ecology

Widely adapted, *S. sesban* tolerates drought, waterlogging, soil acidity, alkalinity, and salinity [29]. *S. sesban* grows well in the subtropics and in cooler, higher elevation regions of the tropics [6, 30]. It is ideally suited to seasonally flooded environments [6]. It occurs naturally in wet habitats such as lake shores, on muddy river banks, and in seasonally flooded valley bottoms [5]. In Chad, *S. sesban* is becoming rare in N'djamena but can be found in the rainy season, while in winter, the species occurs mainly in ponds (called in Chadian dialect “Bouta”) and on the shores of the Chari and Logone rivers (Ousman B. M., 2024) (unpublished). It also grows in open savannah [31] and dry, semiarid zones [21, 23]. It grows in a wide variety of soils from loose, sandy soils to heavy clays [32, 33]. *S. sesban* has moderate tolerance to frost [34]. *S. sesban* grows well on acidic and infertile soils in a semiarid region of Rwanda [35].

#### 3.1.3. Biophysical Limits

The mean annual growing temperature of *S. sesban* is between 18°C and 23°C (maximum 45°C), and the mean annual rainfall ranges from 500 to 2000 mm [6, 34]. Its altitude ranges between 100 and 2500 m [6, 34].

#### 3.1.4. Pests and Diseases

*Sesbania* spp. is attacked by nematodes, insects, fungi, and viruses [6]. The leaf-eating beetle *Mesoplatys ochroptera* can completely defoliate *S. sesban* leading to mortality. Various caterpillars, *Hymenoptera*, and stem borers attack *S. sesban.* Some potentially destructive root-knot nematodes have been recorded in India on *S. sesban* [6]. *Sesbania* is infected by mild and severe mosaic disease virus, which is transmitted by sap and roots, showing vein clearing and reduction of leaflets. The prevalence of infection with mosaic disease virus ranges from 5 to 20%. *Sesbania* plants grown in vitro with mild mosaic virus inoculation had fewer pods and were very small. The virus inoculated in vitro has great tolerance to dilution (between 1000 and 10,000), resistance to heat (40–60°C), and has longevity in vitro varying between 10 and 14 days [36]. Sileshi et al. [37] conducted a survey in Southern Malawi and found that insects *Anoplocnemis curvipes*, *Aphis fabae*, *Hilda patruelis*, *Megalurothrips sjostedti*, *Mylabris dicincta*, *Nezara viridula*, and *Ootheca* spp. have the potential to become pests of *S. sesban*.

#### 3.1.5. Morphological Description

**Figure 4:**
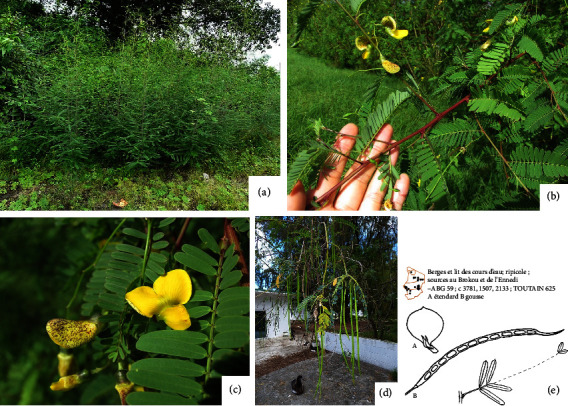
(a) *S. sesban* tree or shrub [38]. (b) *S. sesban* leaves and stem [39]. (c) *S. sesban* flowers [39]. (d) *S. sesban* seedpods [39]. (e) Drawing of seedpods and leaves [14].

*S. sesban* is a soft, slightly woody, and short-lived shrub or small tree reaching 3-4 m tall and is broadleaved and seed propagated (Figure 4(a)) [21, 24, 27, 38, 40–43] (Ousman B. M., 2024) (unpublished). Partey et al. [30] described *S. sesban* as a narrow-crowned, deep-rooting, single- or multi-stemmed shrub or small tree, 1–5 m tall (Figure 4(a)). Shun-ching [44] reported that in Taiwan, *S. sesban* measures approximately 4-5 meters in height, after six months with a diameter of up to 12 cm. César and Chatelain [14] also mentioned that *S. sesban* is a tall shrub plant measuring 3-4 m (Figure 4(a)). The average diameter growth measured in basal circumference ranges from 16 to 28 cm. Branches have opposite pairs in a straight line, with points that look like hairs (Figure 4(b)) [39]. There are at least 20 pairs of leaves that cross one by one each 180° from the previous one and forming a cone that gradually closes (Figures 4(b) and 4(c)) [14, 39]. These leaves are odd-pinnate with one pair of leaflets at the base having large, irregularly lobed terminal leaflets (Figures 4(b) and 4(c)). The flowers are yellow and are arranged in clusters forming from 2 to 20 flowers and almost 20 cm long. The filament sheath is 9–13 mm and yellow-purple speckled and, in rare cases, is pure yellow (Figures 4(b) and 4(c)) [14, 39]. The plant is glabrescent or glabrous (Figures 4(a)–4(c)) [5, 14, 15]. Five to seven seedpods are grouped together in the form of grapes (Figure 4(d)) [14]. Seedpods are subcylindrical, light green just after formation, and yellow in color when maturing, straight or slightly curved, up to 30 cm long and 5 mm wide, containing 10–50 seeds (Figure 4(d)) [14, 39]. Drawings of seedpods and leaves are presented in Figure 4(e) [14]. Soaking the seeds in water for a few days is sometimes required to make them germinate [15].

We note that the botanical missions carried out on *S. sesban* date from 1968 by the botanist, Léonard [19], and from 2019 by the botanists, César and Chatelain [14]. Léonard collected this species in 1968 in the region of Lake Chad [19] (Figure 1).

### 3.2. Uses of *S. sesban*: Medicinal Use, Biological Activity, and Phytochemistry

#### 3.2.1. Medicinal Use and Biological Activity

In sub-Saharan Africa, the use of plant resources for therapeutic/medicinal, agricultural, and other purposes is common, hence the need for intervention in protecting and enhancing these resources [45]. The local population in Chad is aware of the importance of the species *S. sesban* and benefits from its use for medicine, for improving land cover and soil fertility, for feeding and shading livestock and for wood [46, 47] (Ousman B. M, 2023) (unpublished). Abdelgawad et al. [48] conducted a holistic overview on *S. sesban* leaves and their phytoconstituents and pharmacological activities or effects [49] and presented that *S. sesban* leaves exhibited several therapeutic potentials such as antioxidant, antimicrobial, antiviral, anthelmintic, molluscicidal, antifertility, anti-inflammatory, antidiabetic, antihyperlipidemic, anticancer, antianxiety, and mosquito repellant properties. More detail is provided in the subsection text as follows.

#### 3.2.2. Antioxidant Activity

Mani et al. [40] evaluated *the in vitro* antioxidant and antimicrobial activities of *S. sesban* leaves' ethanolic extracts. The phytochemical screening reports the presence of saponins, tannins, phenolic compounds, and flavonoids. The antioxidant activity of ethanolic extracts was demonstrated by the DPPH (diphenyl-2-picryl hydrazyl) radical scavenging test, which shows a remarkable scavenging activity depending on the dose of 100 *µ*g/ml. The reducing capacity increased with the increasing concentration of the sample. When the 100 *µ*g/ml ethanol extract was found, the active free radical scavenging activity increased from 16.71% (20 *µ*g/ml) to 76.25% (100 *µ*g/ml). This reducing power serves as a significant indicator of antioxidant activity.

Kathiresh et al. [41] found further evidence of *S. sesban*'s antioxidant activity. They extracted anthocyanin compounds, total phenol, and flavonoids from *S. sesban* flower petals using methanol and acidified methanol extracts. The anthocyanin content confirmed by ferric chloride and aluminium chloride tests was used for analysing the antioxidant and antimicrobial properties. The total anthocyanin content obtained from the methanol and acidified methanol extracts was 0.38 mg/100 g and 0.28 mg/100 g, respectively. The antioxidant activity of acidified methanol extracts using the hydrogen peroxide test showed high scavenging activity of 84% at lower concentration (1 mg/ml) along with the standard butylated hydroxytoluene (37.65%).

#### 3.2.3. Antimicrobial Activity

Kathiresh et al. [41] found further evidence of *S. sesban*'s antimicrobial activity. The antimicrobial property of *S. sesban* flower extract was explained by the zone of inhibition occurring around the wells containing different concentrations of the extracts in the disc diffusion assay. This antimicrobial activity of the samples after 24 hours showed that the zone of inhibition was found in Gram-positive bacteria (*Staphylococcus aureus* (1 mg) and *Staphylococcus saprophyticus* (12.5 mg)), whereas there was no inhibition in Gram-negative bacteria (*Escherichia coli Klebsiella pneumoniae Klebsiella oxytoca*, *Proteus vulgaris*, *Streptococcus pyogenes*, *Pseudomonas aeruginosa*, and *Enterococcus faecalis*).

Ahmed et al. [50] found further evidence of *S. sesban*'s antimicrobial features justifying the use of the bark to treat a number of ailments in Bangladesh. They investigated the phytochemical screening of ethanol, ether, and chloroform extracts of the plant bark and found the presence of carbohydrates, flavonoids, steroids, alkaloids, tannins, and saponins. The antimicrobial activity using the disc diffusion assay after 18 hours showed that the chloroform extract (at the dose of 250 *μ*g/ml and 500 *μ*g/ml) and the ethanol extract (500 *μ*g/ml) of *S. sesban* bark inhibited all bacteria used: 5 Gram-positive bacteria (*Staphylococcus epidermidis*, *Streptococcus pyogenes*, *Staphylococcus aureus*, *Enterococcus faecalis*, *Bacillus subtilis*) and 9 Gram-negative bacteria (*Shigella boydii*, *Shigella flexneri*, *Shigella sonnei*, *Shigella dysenteriae*, *Escherichia coli*, *Proteus vulgaris*, *Erwinia amylovora*, *Klebsiella pneumonia*, *Pseudomonas aeruginosa*). In a disk diffusion assay, 250 *μ*g/ml of the ethanol extract of bark inhibited all microorganisms except for *Proteus vulgaris* and *Enterococcus faecalis.* The ether extracts (both 250 *μ*g/ml and 500 *μ*g/ml) of *S. sesban* bark inhibited all microorganisms except for *Proteus vulgaris*.

#### 3.2.4. Anthelmintic Activity

Ibrahim [51] examined the anthelmintic activity in vitro of *S. sesban* leaves aqueous extract (0.25–50 mg/ml) using the free-living rhabditid nematode, *Caenorhabditis elegans*. A considerable amount of anthelmintic activity was demonstrated by extracts of *S. sesban* leaves (2.5 mg/ml) when the percentage mortality of *Caenorhabditis elegans* nematodes was 30% and 96% after 2 h and 6 h treatments, respectively. Seed extracts introduced percentage mortality of 5.6–25.5% at concentration levels of 0.25–50 mg/ml. They concluded that *S. sesban* showed the highest anthelmintic effect on *Caenorhabditis elegans* survival at concentration levels 2.5 mg/mL, and the median lethal concentration LC_50_ values of *S. sesban* leaves were the most effective compared to other plant species studied. The LC_50_ attained 8.0 mg/mL at a minimum effective concentration of 2.5 mg/ml.

In the study of Limsay et al. [52], the hydroethanolic and aqueous leaf extracts of *S. sesban* (at a concentration of 5 and 10 mg/mL and 30 mg/mL for hydroethanolic leaf extract and ethyl acetate fraction) were evaluated for their anthelmintic activity *in vitro*, against *Moneizia expansa* and *Paramphistomes* using a Petri-dish method, with fenbendazole as the control. The methanolic extract was also evaluated in rats for its anthelmintic effect *in vitro* against two intestinal parasites *Hymenolepis diminuta* (in rats), a cestode, and *Syphacia obvelata* (in mice), a nematode, with praziquantel and albendazole as reference drugs. Interesting results were obtained for this anthelmintic effect with the inhibition zone of 15.17 mm against *Syphacia obvelata*-mice at the concentration dose of 30 mg/mL of hydroethanolic leaf extract and 7.56 mm against *Moneizia expansa* at the concentration dose of 5 mg/ml of aqueous leaf extract [53, 54].

Numerous studies across the world have shown that *S. sesban* has considerable potential for combating diseases and improving health. Kamel et al. [55] reported that the administration of *S. sesban* leaves' methanol extract to infected mice exhibited a moderate antischistosomal effect (against the parasite *Schistosoma mansoni* which infected mice). The results suggest that the administration of *S. sesban* has antischistosomal properties, hence ameliorating liver function.

#### 3.2.5. Antibacterial Activity

Mythili and Ravindhran [42] observed the presence of alkaloids, flavonoids, phenols, and phytosterols, fixed oil and gum in the phytochemical analysis of methanol, and ethanol extracts from *S. sesban* in India. The authors tested the biological screening effects of *S. sesban* methanol stem extract on ten bacterial species (Gram + pathogens: *Staphylococcus aureus* (ATCC 25923), *Enterococcus faecalis* (ATCC 29212), *Escherichia coli* (ATCC 25922), *Bacillus* subtilis (ATCC 441), and Gram−pathogens: *Salmonella typhi* (MTCC 733), *Erwinia amylovora* (MTCC 2760), *Proteus vulgaris* (MTCC 1771), *Pseudomonas aeruginosa* (MTCC 424), *Klebsiella pneumoniae* (ATCC 15380), and *Shigella dysenteriae* (MTCC 5151)). The results showed a highly significant activity against the bacteria *Erwinia amylovora* with 17.25 mm in diameter followed by *Escherichia coli* with 16 mm in diameter at 250 *µ*g/ml of the extract. In most of the bacteria examined, a better zone of inhibition was obtained at 250 *µ*g/ml and 500 *µ*g/ml of the extract. When compared to the standard flavonoid quercetin, the plant extract showed a substantial amount of inhibition in the case of *Bacillus subtilis* (15.5 mm) (500 *µ*g/ml), *Escherichia coli* (16 mm) (250 *µ*g/ml), *Enterococcus faecalis* (12.75 mm) (250 *µ*g/ml), *Erwinia amylovora* (17.25 mm) (250 *µ*g/ml), and *Shigella dysenteriae* (10.25 mm) (500 *µ*g/ml).

In Sudanese folk medicine, the leaves and fruits of *S. sesban* were found to treat sore throat and gonorrhoea in the study of Elegami et al. [56]. They investigated whether the leaves and fruits of *S. sesban* have antibacterial activity. They found that methanol extracts at the concentration dose of 100 mg/ml (0.1 ml/cup) were effective against pathogen bacteria used, namely, *Bacillus subtilis* NCTC 8236, *Staphylococcus aureus* NCTC 6447, *Escherichia coli* NCTC 8196, and *Pseudomonas aeruginosa* NCTC 6750.

#### 3.2.6. Antifungal Activity

Mythili and Ravindhran [42] tested the biological screening effects of *S. sesban* methanol stem extract on five infectious fungal species (*Aspergillus fumigatus*, *Colletotrichum gloeosporioides*, *Curvularia lunata*, *Fusarium oxysporum*, and *Verticillium glaucum*) using the disc diffusion assay. The fungi *Curvularia lunata* and *Fusarium oxysporum* were inhibited completely by *S. sesban* methanol stem extract at the dose of 100 *µ*g/ml and 500 *µ*g/ml. For *Aspergillus fumigatus*, *Curvularia lunata*, and *Verticillium glaucum*, a higher degree of inhibition was obtained with the dose of 500 *µ*g/ml. They concluded that the stem extracts of *S. sesban* possess a broad spectrum of activity against common bacterial and fungal diseases in the region of Coimbatore in India.

Ahmed et al. [50] evaluated the antifungal activity of *S. sesban* bark against 7 fungi (*Trichophyton rubrum*, *Microsporum fulvum*, *Candida albicans*, *Curvularia lunata*, *Aspergillus fumigatus*, *Fusarium oxysporum*, and *Saccharomyces cerevacae*) using the disc diffusion assay. The result showed that the highest zone of inhibition was 14.2 mm against *Fusarium oxysporum*. Minimum inhibitory concentration (MIC) of these extracts was determined by the broth macrodilution assay. After 12 hours, the MIC of the extracts (ethanol, ether, and chloroform) was obtained at a higher concentration (8000 *µ*g/ml) than the extract content in the disc (250 *µ*g/ml and 500 *μ*g/ml).

#### 3.2.7. Anti-Inflammatory Activity

In India, many people use *S. sesban* leaves to relieve rheumatic pain and the biochemical evidence supporting this is clear [43]. Crude saponins (containing triterpenoids and steroids) extracted from *S. sesban* leaves showed an anti-inflammatory effect on experimental-induced rats and mice [43]. Rats pretreated with saponins significantly decreased (*p* < 0.01) the carrageenan-induced paw edema by 59% at a higher dose of 500 mg/kg, 3 h after the injection of the noxious agent. Rats pretreated with saponins significantly inhibited (*p* < 0.01) the histamine-induced rat paw edema by 38.41 and 43.02% at the dose of 250 and 500 mg/kg, respectively. The test of cotton pellet granuloma in rats showed that saponins (500 mg/kg) inhibited the formation of fibroblasts by 38.17% which was comparable with that of standard diclofenac sodium (44.32%). In the oxazolone-induced delayed hypersensitivity test, saponins (500 mg/kg) showed maximum inhibition (69.68% after 22 days) of ear edema comparable to the standard drug which gave 73% inhibition after 22 days. Saponins therefore showed significant activity in the acute phase of inflammation in the *in vivo* and *in vitro* models at the oral dose of 500 mg/kg body rats' weight when compared to the control and standard drug.

Similarly, Shaikh et al. [32] evaluated the anti-inflammatory activity of *S. sesban* leaf extracts of petroleum ether (60–800), chloroform, and methanol. The acute toxicity study of extracts of leaves of *S sesban* showed 50% mortality at a dose 2500 mg/kg. Hence, 1/10th of the same dose for all these extracts was taken as a therapeutic dose, i.e., 250 mg/kg. The methanolic extract showed a significant anti-inflammatory activity reducing paw edema (the dose of 250 mg/kg administered reduced 45.34% of the reduction within three hours) compared to the control group, carrageenin and ibuprofen. Petroleum ether (60–80°) extract and chloroform extract showed comparatively less reduction in paw edema volume. Thin layer chromatography using the solvent system toluene:chloroform:methanol (1 : 1 : 0.8) followed by the column chromatography for separation and isolation of the constituents of the methanolic extract showed three separated constituents: Constituent I (fluorescent green color), Constituent II (pink color), and Constituent III (fluorescent green color). Constituent II showed a significant reduction in paw edema (at the dose of 250 mg/kg) meaning significant anti-inflammatory activity after three hours (with a percentage of 54.06) as compared to the standard. The preliminary phytochemical investigation *S. sesban* extracts showed the presence of the following active principles: sterols, saponins, flavonoids in methanol extracts; fats and oil in petroleum ether (60–80°) extracts; and sterols, alkaloids, and flavonoids in chloroform extracts. The study did not show any approximate percentage, while it showed simply the presence or absence of these active principles obtained.

Ahmed et al. [50] found further evidence of *S. sesban'*s cytotoxic features, and the cytotoxicity activity was investigated by the brine shrimp lethality bioassay to determine the percent mortality nauplii caused by the test extracts. The LC_50_ (lethal concentration in half) values of ethanol, ether, and chloroform extracts of bark were found to be 1280, 640, and 320 *µ*g/ml, respectively, after 24 hours.

#### 3.2.8. Antidiabetic Activity

Pandhare et al. [57] evaluated the aqueous leaf extract of *S. sesban* for its antidiabetic potential in normal and streptozotocin (STZ)-induced diabetic rats. Doses of 250 and 500 mg/kg body weight per day for 30 days were administered to normal and streptozotocin-induced diabetic rats. The aqueous leaf extract administered (250 and 500 mg/kg/day) to streptozotocin-induced diabetic rats (compared to the antidiabetic drug glibenclamide (0.25 mg/kg body weight) indicated a significant increase in the body weight, liver glycogen, serum insulin, and high-density lipoproteins cholesterol levels and decrease in blood glucose, glycosylated haemoglobin, total cholesterol, and serum triglycerides. Finally, the study concluded that the aqueous leaf extract of *S. sesban* has beneficial effects in reducing elevated blood glucose levels and the lipid profile of streptozotocin-induced diabetic rats but has no effect on normal rats.

Manjusha et al. [58] found that *S. sesban* root extract may have a hypoglycaemic potential for treating type 2 diabetes. Their results show that the doses (250, 500, and 1000 mg/kg) of *S. sesban* root extract administered orally to normal and streptozotocin (STZ)-induced type-2 diabetic mice caused a marked decrease in fasting blood glucose in STZ-induced type-2 diabetic mice. *S. sesban* root extract decreased the cholesterol, triglyceride, urea, and creatinine levels and increased insulin, high density lipoproteins, cholesterol, and total protein levels.

#### 3.2.9. Antinociceptive Activity (Analgesic)

Nirmal et al. [59] investigated wood's antinociceptive agents that are compounds capable of diminishing pain without negative effects on consciousness or without producing anaesthesia [60]. The antinociceptive activity was determined by hot plate and acetic acid-induced tests. Doses were selected on the basis of a toxicity study, and mice were divided into 18 groups of 6 animals each. The experiment was terminated 20 seconds after their placement on the hot plate to avoid damage to the paws. Petroleum ether, chloroform, and ethyl acetate extracts (50 and 100 mg/kg) showed significant results just 30 minutes after treatment, while their action was blocked by the opioid antagonist, naloxone (1 mg/kg). The involvement of opioid receptors (transmembrane neurotransmitters) was revealed by giving the extracts after an opioid antagonist (naloxone) (1 mg/kg). The mechanism of the analgesic effect of the extracts of *S. sesban* wood could probably be due to blockage of the effect or release of endogenous substances that excite pain nerve endings [59]. They concluded that petroleum ether, chloroform, and ethyl acetate extracts of wood showed potent antinociceptive activity, while naloxone blocked the antinociceptive activity of the extracts by inducing opioid receptors.

#### 3.2.10. Control of Fertility

Some studies suggest that *S. sesban* may have potential as an ingredient in contraceptives. Shiv [61] studied the effect of *S. sesban* seed powder in female albino rats to evaluate its effects on genital organs and fertility. In the results, rats in the control group did not show any change in body weight and genital organ weight. The dose 100 mg/kg for 30 days of administration had no deleterious effect on ovarian tissues, whereas the 250 mg/kg dose severally affected the ovarian structure, mature follicles underwent atresia, some developing follicles showed lysis of ova, and the stroma was compact with poor vascularity. However, the genital organ weight was reduced significantly (*P* < 0.05) after the treatment at 250 and 400 mg/kg/day doses for 30 days. The dose of 250 mg/kg reduced endometrial height and size of the uterine glands. The administration of 400 mg/kg for 30 days caused a great reduction in endometrial height and uterine glands. The control group of rats showed normal fertility; all became pregnant and showed a good number of implants, whereas the dose 100 mg/kg dose showed pregnancy and reduction of implants. The doses 250 and 400 mg/kg showed 100% antifertility activity, and no implants were recorded in the uterus of these rats on the 10th day of pregnancy. The experiment showed that *S. sesban* seed powder inhibits ovarian function, changes the uterine structure, and prevents implantation and, thus, controls the fertility of female albino rats [61].

In another study, Das et al. [62] isolated from *S. sesban* roots extracts an active principle oleanolic acid 3-beta-D-glucuronide (OAG) which is suggested to have a potent spermicidal activity. In the experiment, the dose of the minimum effective concentration (MEC) of OAG was 50 mcg/mL after one hour of the treatment and induced 100% immobilization of the sperm. More than 97% of the OAG-treated sperm lost their hypoosmotic swelling responsiveness in a dose-dependent manner. Transmission electron microscopy and sperm membrane lipid peroxidation revealed that OAG affected the sperm membrane integrity. All observations in the experiment clearly demonstrated that OAG has very strong antifertility activity and other properties that qualify the agent to serve as an active ingredient of vaginal contraceptives [62].

#### 3.2.11. Central Nervous System Stimulant

The aqueous extract of *S. sesban* bark has a potential central nervous system (CNS) stimulant effect [63]. The investigation of CNS stimulant activity was carried out on albino mice, and caffeine was used as a reference drug. The animals receiving the treatment were divided into three groups: Group I served as control and was treated orally with vehicle (normal saline), Group II served as the standard, and caffeine 30 mg/kg was given, and Group III received aqueous extract of *S. sesban* bark at the dose of 400 mg/kg. In the elevated plus maze experiment, the animals received the treatment 45 min before the start of the session. At the beginning of the session, a mouse was placed at the centre of the maze, its head facing the closed arm. It was allowed to explore the maze for 5 minutes. The time spent in the open arms, percent entries in the open and closed arms, and total entries were recorded. An entry was defined as the presence of all four paws in the arm. Naik et al. [63] concluded that the crude aqueous extract at a dose of 400 mg/kg after 48 hours showed significant central nervous system CNS stimulant activity in comparison to the control group, and the results were comparable to the activity shown by the reference drug.  Table 2 summarizes *S. sesban's* medicinal use, the biological activity, the plant parts used and optimal solvents, the dosage regimen and its corresponding concentrations, and the active constituents.

#### 3.2.12. Molluscicidal Activity

*S. sesban* leaf extracts showed molluscicidal activity. In the study of [64], the effects of sublethal concentrations of methanol extract of *S. sesban* leaves on the survival rate, egg laying of *Bulinus truncatus* snails, hatchability of their eggs, infection rate with *Schistosoma haematobium* miracidia, cercarial production, and certain physiological parameters of treated snails were studied. In the results, after 24 hours of exposure, the sublethal concentrations of the tested plant extract (LC_0_ = 1.8 ppm, LC_10_ = 8 ppm, LC_25_ = 14, LC_50_ = 18 ppm, and LC_90_ = 31 ppm) caused a considerable reduction in survival rates; egg production of *Bulinus truncatus* snails; and hatchability of eggs as well as in the infectivity of *Schistosoma haematobium* miracidia to the snail. The longevity of *Bulinus truncatus* snails exposed continuously to sublethal concentrations of methanol extract of *S. sesban* decreased from LC_0_ = 22.5 ± 6.2 days to LC_10_ = 11.8 ± 4.2. The death rate of *Bulinus truncatus* snails in groups treated with LC_0_ was highly significant as compared with those in groups treated with LC_10_ and LC_25_ (*p* < 0.01). A reduction in cercarial production per snail and the period of cercarial shedding were also observed. Glycogen level, protein content, and the activities of hexokinase (HK), pyruvate kinase (PK), and lactate dehydrogenase (LDH) showed a decrease in soft tissues when compared with the control group. They concluded that the application of a sublethal concentration of methanol extracts of *S. sesban* leaves may be helpful in snail control as it interferes with the snails' biology and physiology.

Furthermore, in the study of [65], the molluscicidal activity against snail species *Biomphalaria alexandrina* infected with *Schistosoma mansoni* was investigated. *Biomphalaria alexandrina* species was treated with aqueous extracts of *S. sesban* leaves. The extracts significantly lowered the infection rate of the snail. Exposure of snails for 4 weeks to LC_10_ and LC_25_ of S*. sesban* leaves (dry powder) considerably suppressed their fecundity and the reproduction rate. The reduction rate of reproduction for the exposed snail to LC_25_ of *S. sesban* was 76.4%. Infection rates of snails treated during miracidial exposure with LC_10_ of *S. sesban* was 52.2% compared to 92.6% for the control group (*p* < 0.01). Snails exposed to LC_25_ of *S. sesban* leaves extracts showed a reduction of the duration of cercarial shedding and cercarial production/snail with a value of 223.2 cercariae/snail compared to 766.3 cercariae/infected control snail (*p* < 0.01). It is concluded that LC_25_ of *S. sesban* leave aqueous extracts negatively interferes with biological and physiological activities of *Biomphalaria alexandrina* snails; consequently, it could be effective in interrupting and minimizing the transmission of *Schistosoma mansoni* [65]. Saponins are some of the secondary metabolites that are synthesized by many plants [66]. The molluscicidal activity of *S*. *sesban* can be attributed to saponins whose mode of action is believed to cause cell membrane rapture causing water and ions to flow uncontrollably into and out of the cell. This causes the cell to lose integrity leading to the death of the snails [49].

#### 3.2.13. Phytochemistry of *S. sesban*

*S. sesban* has different chemical compounds that are, once extracted, very useful for treating diseases such as antibacterial and antioxidant agents, for manufacturing drugs and organic or chemical supplements. They are also useful for manufacturing biological manure [41, 67]. The phytochemical screening test of different leaf extracts of *S. sesban* (methanol, chloroform, and petroleum ether (60–80°)) in the study of [32] revealed the presence of sterols, saponins, flavonoids, alkaloids, fats and oils, proteins, sterols, anthraquinone glycosides, gums, and miscellaneous compounds. Carbohydrates, vitamins, amino acids, tannins, and saponins, and glycosides are also detected in the screening test of the aqueous extracts in the study of [57]. Ahmed et al. [50] studied the chemical screening of *S. sesban* bark, and the results indicated the presence of carbohydrates, flavonoids, steroids, alkaloids, tannins, and saponins in the ethanol, ether (diethyl ether), and chloroform extracts (95% each one).

Samajdar and Ghosh [68] reported from different studies that the preliminary phytochemical screening of *S. sesban* uncovered the presence of triterpenoids, starches, vitamins, amino acids, proteins, tannins, saponins, glycosides, and steroids. Blossoms contain cyanidin and delphinidin glucosides. Dust and dust tubes contain alpha-ketoglutaric, oxaloacetic, and pyruvic acids. Leaf and unit contain campesterol cholesterol, beta-sitosterol, triterpenoids, proteins, and tannins. Bark and stem contain glucose, fructose, erythritol, arabinitol, and myo-inositol. Different kinds of lignins are made out of guaiacyl, syringyl, and P-hydroxyphenylpropane building units and furthermore antitumor vital kaempferol disaccharide [68–70]. Sterols and triterpenes are detected in petroleum ether and chloroform extracts of *S. sesban* wood and flavonoids in ethyl acetate extract in the study of [59]. Carbohydrates, alkaloids, phytosterols, saponins, glycosides, and phenolic compounds are detected in petroleum ether, chloroform, and aqueous extracts of *S. Sesban* bark [63]. In the study of [42], the phytochemical analysis of the methanol and ethanol extracts of both stem and root of *S. sesban* revealed the presence of alkaloids, carbohydrates, proteins, phytosterol, phenol, flavonoids, fixed oil, and gum. The leaf extract showed the presence of alkaloids, carbohydrates, protein, phytosterol, flavonoids, and fixed oil.

Leaves of *S. sesban* are used as supplementation for growth and reproduction performance in 30 male Ethiopian highland sheep and 25 East African goats [71]. In this study, many chemical compositions in *S. sesban*' leaves for the feed ingredient were detected among them: dry matter, crude protein, gas production, ash, neutral detergent fibre, acid detergent fibre, neutral detergent fibre-bound nitrogen, soluble proanthocyanidins, quercetin, and saponin [71, 72]. Anthocyanins, phenols, and flavonoids are identified in methanol and acidified methanol extracts of *S. sesban* flower petals [41]. The oleanolic acid 3-*β*-D-glucuronide has been isolated and evaluated from the root extracts of *S. sesban* [62]. In the chemical study of Abdelgawad et al. [48], many phytochemical compounds of *S. sesban* leaves were presented and had a variety of essential metabolites belonging to different chemical classes including steroids, triterpenoids, saponins, flavonoids, coumarins, lipids, and other miscellaneous compounds. Details of phytochemical compounds extracted from part of the species *S. sesban* with the extraction solvent are shown in Table 3.

### 3.3. Ethnomedicinal Uses of *S. sesban*

#### 3.3.1. Treating Breast Cancer, Edemas, and Wounds

Healers in Chad use the leaves and bark of *S. sesban* alone to treat breast cancer, edemas, and wounds. Breast cancer is treated in traditional medicine by macerating the leaves for 48 hours or using an infusion of root and bark. After macerating the leaves, the juice obtained was drunk in the morning and evening. To treat edema and swollen glands, the leaf powder is mixed with oil and then applied to the body until cure. In Chad, traditional healers often sell *S. sesban* formulations from leaves and bark to patients in the form of syrup or powder to treat breast cancer, edema, and wounds in the same manner as previous use. Healers sold such medicine about 360 times per year, earning an average of $1.74 per sale. Annual revenue thus amounted to about $US 625$/year (Ousman B. M., 2024) (unpublished).

#### 3.3.2. Treating Livestock Diseases

*S. sesban* is also used in treating livestock diseases. Harun-or-Rashid et al. [73] reported that the leaves of *S. sesban* are used in Bangladesh for the treatment of cattle diseases. The leaves are administered orally to treat the retention of urine in cows, goats, and buffaloes. Similarly, Rahmatullah [74] conducted an ethnoveterinary survey among selected villages of Bagerhat district in Bangladesh and documented that *S. sesban* leaves and stems are used topically to treat pain arising from pox of cattle. The leaves are dried in sunlight and then spread over the bodies of cows, goats, or buffaloes. At the same time, the bodies of cows, goats, or buffaloes are brushed with stems and leaves [74]. Sori et al. [75] reported that pastoralists of Borana district in the Southern Ethiopia use *S. sesban* root and bark to treat mastitis in order to control the disease of the livestock. The infusion of root and bark is topically used for the treatment of mastitis.

#### 3.3.3. Treating Malaria

Chinsembu [76] in Zambia and Rasoanaivo et al. [77] in Madagascar reported, respectively, that the vapour of *S. sesban* leaves obtained from boiling is inhaled two times a day for three days, and a drinking decoction of aerial parts is used to cure malaria.

#### 3.3.4. Mosquito Repellant

Samajdar and Ghosh [68] reported that *S. sesban* leaves are used as mosquito repellents in India for livestock. The preparation method is to wash the bodies of animals with water leaf extracts until cure [68]. The leaf decoction is used for cattle drench to repel tsetse flies in India [68].

#### 3.3.5. Demulcent, Anthelmintic, Purgative, Anti-Inflammatory, and Treating Eczema

Abdelgawad et al. [48] mentioned that *S. sesban* leaves have been traditionally used as anthelmintic, demulcent, purgative, and anti-inflammatory agents in the treatment of eczema, in addition to its agricultural uses.  Table 4 shows the ethnomedicinal use of *S. sesban*, plant part used, dosage regimen, and mode of preparation.

### 3.4. *S. sesban* Use for Soil Improvement in Agriculture and in Cropping Systems

Land degradation and declining soil fertility are critical problems impacting livelihoods in many parts of Africa [3].

#### 3.4.1. Inducing Root Nodules of *Rhizobium* Strains and Fixing Nitrogen on the Soil to Improve It

In Chad, the local population uses *S. sesban* to enrich soil fertility of the cited soils for increasing yields of crops such as *Oryza* sativa L., *Zea mays* L., *Sorghum bicolor* L., and *Cenchrus americanus* (pearl millet) (Ousman B. M., 2024) (unpublished).

In México, Bashan et al. [8] have demonstrated that native leguminous trees such as *S. sesban* are essential to ensure the revegetation of eroded desert lands and restoration of severely eroded soil by fixing nitrogen, resisting salt and drought, and producing high biomass under desert conditions at the southern limit of the Sonoran desert in agricultural and agroforestry systems. The population plants the leguminous trees such as *S. sesban*, *Prosopis articulata*, *Parkinsonia microphylla*, and *Parkinsonia florida* and inoculates with growth promoting bacteria *Azospirillum brasilense*, *Bacillus pumilus*, a native fungus *Arbuscular mycorrhizal* (AM), and small quantities of compost. A high density of these leguminous trees with shrubs and trees was obtained in these severely eroded soil areas with a remarkable degree of revegetation and more stabilized soil with a high volume of organic matter.

Samajdar and Ghosh [68] mentioned that *S. sesban* is appreciated for its nitrogen-fixing quantities and as a windbreak on farms in India.

Abbas et al. [23] found in Egypt that *S. sesban* intercropped with some annual grasses (barley, pearl millet, and Rhodes-rye and Sudan grasses) and inoculated with rhizobia improved the quality and quantity of field forage crops. They also found that intercropping improved the productivity of nonlegumes, in particular barley mixed with the legume *S. sesban*, and the calculated N-transfer from legumes to nonlegumes ranged from 20 to 70 kg·N/ha. They concluded that intercropping of forage grasses with legumes is economic and has a high environmental return under the semiarid conditions of Egypt and that *S. sesban* performs better when intercropped with Sudan grass.

Sobere [78], Bala et al. [79], and Sharma et al. [80] reported in the same way that *Rhizobium* strains induce root nodules and fix nitrogen from the air in symbiosis with *S. sesban.*

Curasson [7] and Rochester et al. [81] reported that *S. sesban* is cultivated in rotation with cotton in Sudan and Australia, and it may enhance soil fertility and improve soil conditions. *S. sesban* yields are reported to range from 28 to 35 tons/hectare after three years of growth [7].

Balaisubramanian and Sekayange [35] reported on an experiment in a semiarid site in Rwanda with *S. sesban* grown as a hedge spaced 5 meters apart in cultivation with beans (*Phaseolus vulgaris* L.), sorghum (*Sorghum bicolor* L.), maize (*Zea mays* L.), and sweet potato (*Ipomoea batatas* L.) that the produced foliar biomass was 1.78 and 0.59 t/hectare, respectively, for 1983/84 and 1985/86. The wood produced was 0.27 and 0.28 t/hectare for 1983/84 and 1985/86, respectively. In addition, it allowed the production of nutrients for the soil of 25.6 kg of nitrogen/ha, 1.4 kg of phosphate/ha, 14 kg of potassium/ha, 16.2 kg of calcium/ha, and 4.4 kg of magnesium/ha, significantly improving soil fertility.

Other studies have found that *S. sesban* improves soil fertility. Bakhoum et al. [82] reported that planting nitrogen-fixing trees such as *S. sesban* which is effective in increasing soil productivity.

Nigussie and Getachew [83] and Degefu et al. [18] reported that *S. sesban* can restore eroded soil by fixing nitrogen in the soil. Mengistu et al. [84] reported on the use of *S. sesban* as green manure in Ethiopia. *S. sesban* biomass decomposes rapidly due to its soft plant structure and high N content, and it provides nutrients to the soil and other plants. It is used for the improvement of fallows, mixed cropping, relay cropping, and biomass transfer [85]. *S. sesban* helps restore and enhances soil fertility by drawing up nutrients from lower soil layers and then adds nutrients to the soil in litter fall [85].

Chandra et al. [86] harvested the biomass of *S. sesban* accessions 20 days after sowing and used it as a green manure crop in a rotation of rice-rice-mustard. They demonstrated that *S. sesban* can be grown and harvested in a very short period and still be useful for adding organic matter to the soil. They also pointed out that the decomposability, organic matter accumulation, and N_2_-fixing ability of *S. sesban* biomass make it a suitable cultivar for poor, nutrient-deficient soils [87].

In intercropping and alley cropping, agricultural crops are grown simultaneously with a long-term tree crop to provide annual income while the tree crop matures. Muimba-Kankolongo [88] reported that intercropping sweet potato with *S. sesban* improves the yield of the crop. In the same way, intercropping *S. sesban* with rice and annual grasses in semiarid conditions helps manage weeds and optimize the yield of dry-seeded rice [23, 89]. Singh et al. [89] concluded that the application of wheat residue mulch at 4 t/ha and *S. sesban* intercropped for 30 days were equally effective in controlling weeds associated with dry-seeded rice. Economic analysis showed that *S. sesban* was as effective as mulch in realising higher economic returns for dry-seeded rice yields during 2003 and 2004.

*S. sesban* also has considerable potential in saline environments where many plants cannot grow. In southern Morocco, agriculture systems are limited by the lack of water resources and salinization of surface and underground freshwater sources. The National Institute of Agronomic Research (INRA) has become interested in the adaptation of *S. sesban* to saline environments and its contribution to improving food and fodder production in desert areas. INRA scientists have successfully introduced *S. sesban* in a saline environment for these purposes in the region of Laâyoune [90]. Bala et al. [91] also found that biological nitrogen fixation can be significantly increased by inoculating tree legumes such as *S. sesban* with salinity-tolerant rhizobia under saline conditions.

#### 3.4.2. Tolerating High-Salinity Soil (Up to 20%)

Some authors have reported on the ecological services of the species such as nodulation and its use in intercropping. Nohwar et al. [92] found that *Rhizobia* species isolated from *S. sesban* root nodules growing in different areas of Mumbai, India, have a capacity to adapt in high salinity (up to 20%) zones and have pH tolerance. They claim that these *Rhizobia* species from *S. sesban* are therefore suitable to be used as biofertilizers in unfavourable environmental conditions for legume cropping and could also help reduce the use of chemical fertilizers. They propose that they could be tested in agricultural fields to exploit their natural benefits.

#### 3.4.3. Increasing the Plant Cover

*S. sesban* can play an important role, along with other leguminous species, in land restoration and protection and conservation of indigenous species in Chad [47]. In Chad, the local population, particularly pastoralists, plant *S. sesban* in different types of soils in the South region such as the vertisols, fluvisols, and subarid soils on sand, tropical ferruginous soils, and the arenosol along the banks of Lake Chad [93]. Their objective is to increase the plant cover, to use its wood for construction, and to repel desert encroachment in zones with little vegetation (Ousman B. M., 2024) (unpublished) [7, 23, 47, 90].

#### 3.4.4. Improving Fallows

The International Centre for Research in Agroforestry (ICRAF) has been greatly interested in the role of *S. sesban* in improving fallows, especially in the savannah woodland region of southern Africa [4, 5, 5].

Improved fallows involve planting mainly legume tree/shrub species in rotation with cultivated crops. In Eastern Zambia, Phiri et al. [94] quantified the yield, root zone, soil water balance, and water use efficiency of maize in rotation with 2 years *S. sesban* fallow and of continuous maize with and without fertilizer. The authors found that growing *S sesban* in depleted agricultural fields or on fallow land for 2 or 3 years and then introducing a hybrid maize crop after the fallow period produced encouraging results. *S. sesban* fallow increased grain yield and dry matter production of subsequent maize per unit amount of water used. Average maize grain yields following *S. sesban* fallow and in continuous maize with and without fertilizer were 3, 6, and 1 mg/ha with corresponding water use efficiencies of 4.3, 8.8, and 1.7 kg/mm/ha, respectively. *S. sesban* fallow increased the soil water storage in the soil profile and drainage below the maximum crop root zone compared with conventionally tilled nonfertilized maize [94]. Many farmers in Eastern Province, Zambia, started using *S. sesban* to improve fallows in the late 1990s and early 2000s, but the practice declined for a number of reasons including a reduction in extension support and the introduction of fertilizer subsidies [95].

The details of the use of *S. sesban* for soil improvement in agriculture and in cropping systems are summarized in Table 5.

### 3.5. Use as Feed for Livestock and as Food for Humans

Some studies conducted at the University of Queensland in Australia reported that *S. sesban* has a high nutritive value (28% crude protein) and high dry matter digestibility (86%) [29]. Roothaert and Paterson[96] found in Kenya that *S. sesban* had the highest dry matter digestibility compared to some common fodder tree species such as *Leucaen*, *leucocephala*, and *Calliandra calothyrsus. S. sesban* also had low acid detergent fibre levels and average crude protein content, which gave it a high nutritive value overall. *S. sesban's* seeds contain 39% protein [97]. One hundred gram of dry seeds contained 29−32 g of crude protein, 5-6 g of crude lipid, 16 g of crude fibre, 18-19 g of total starch with 7.2−7.4 g of digestible starch, 4.85–5.95 g of total phenols, 1.97–2.02 g of tannins, 5.05–5.14 g of condensed tannins, 2.35−2.37 g of phytate, and 1.26–1.46 g of saponins [97].

#### 3.5.1. Feed for Livestock

Numerous studies confirm *S. sesban's* high feed value. Access to adequate livestock feed is the main constraint limiting livestock productivity in Africa [98]. *S. sesban* is widely used as a feed across the continent, as evidenced in the following examples. In Chad, livestock keepers cut, carry, and feed the leaves to ruminants. The pods are cut and fed to dairy sheep, goats, and oxen. Leaves and pods are considered high-protein fodder to increase milk productivity (Ousman B. M., 2024) (unpublished). *S. sesban* is sometimes also cultivated in rotation with sugar cane or alone as a feed for sheep and goats, which consume the leaves and young stems [7].

Many studies have been conducted in East Africa to assess animal production characteristics such as growth rates, milk production levels, and fertility when cattle, sheep, and goats were fed tree fodder such as *S. sesban* [99], although its uptake has not been as significant as that of *Calliandra calothyrsus*. However, in Uganda, *S. sesban* is widely grown. In Ethiopia, *S. sesban* is the most important planted fodder tree and is generally grown in home gardens [34]. Smallholders feed it to goats, sheep, and cows. Roothaert and Paterson [96] also reported on a study in which separate groups of local goats with an average initial age and live weight of 8 months and 8.4 kg were allowed to graze daily on the natural ranges for two wet and two dry seasons. They were supplemented at night with sun-dried leaves and small twigs of *S. sesban.* The mean intake was 76 g·day^−1^ per head, and the mean daily live weight gain was 24 g·day^−1^ per head. Mekoya et al. [100] conducted a study in the central highlands of Ethiopia on the effect of the supplementation with *S. sesban* on the milk yield of sheep. They concluded that supplementation of *S. sesban* at 30% of the ratio (0.98% of their body weight) during lactation improved the milk yield of ewes and the growth rate of lambs compared with supplementation with concentrates. *S. sesban* thus has the potential of increasing milk and meat production of sheep and can serve as a substitute ratio to commercial concentrates for resource-poor farmers [100]. Studies conducted by Peters [101] have shown that the leaves of *S. sesban* from Ethiopia are highly nutritious as the crude protein content of the leaves is high (25% to 30% of dry matter), and they contain little tannin and other polyphenols. They reported that *S. sesban* is a useful source of protein for ruminant diets and may prove useful to farmers with livestock and the need for improved fodder [101].

#### 3.5.2. Food for Humans

*S. sesban* is not widely consumed by humans, but Bunma and Balslev [97] reported that there are many uses of *S*. *sesban* for human food. Details of the use of *S. sesban* as feed for livestock and as food for humans are summarized in Table 6.

### 3.6. Other Uses of *S. sesban*

#### 3.6.1. As Fuelwood

Fuelwood availability is a key problem throughout Africa and particularly in Chad [102]. Robert and Abdel-Hamid [102] reported that some important forest trees (including *S. sesban*) will continue to be used as fuelwood for quite some time to come in most sub-Saharan cities and the sustainability of supply is questionable. Research conducted by the World Agroforestry Centre [46] found that *S. sesban* is used in many African countries as a source of fuel and is appreciated because it grows fast, burns well, and can be coppiced.

In western Kenya, Swinkels et al. [103] reported that three-quarters of farmers had *S. sesban* in their cropped fields (mainly maize) and that 20 percent planted it. Its main use was as fuelwood, but farmers also appreciated its contribution to soil fertility [46]. Adelanwa and Tijani [104] in Nigeria and Muimba-Kankolongo [85] reported that the biomass of *S. sesban* can produce wood within just 3–6 months when grown with *Cajanus cajan* (leguminous).

#### 3.6.2. Shade or Shelter and Hedge

*S. sesban* is also used as a shade for humans and their animals, a windbreak, a cover crop, an ornamental plant, and fish poison and for sticks for construction. It is also used for building huts, making charcoal, and preparing gunpowder [7, 15, 68] (Ousman B. M., 2024) (unpublished). *S. sesban* has also grown as a hedge [7].

#### 3.6.3. As Fibre for Ropes and Fishing Nets and as Gum

*S. sesban* is used as a fibre for ropes and fishing nets, and the seeds produce gum [46].

#### 3.6.4. Enhancing Phytoextraction of Heavy Metals from Soil

Gupta et al. [105] found that *S. sesban* may enhance the phytoextraction of heavy metals such as cadmium (Cd), lead (Pb), and zinc (Zn) from artificially contaminated soil by application of ethylene di-amine tetra-acetic acid (EDTA). They reported that *S. sesban* may enhance chemically, by chelate induction, the phytoextraction of the cited heavy metals from the spiked soil through the application of 5 mmol EDTA/kg.

#### 3.6.5. Ecological Service concerning N Nutrition

Dan and Brix [106] evaluated the growth responses of *S. sesban* to NH_4_^+^ (about 70 mg/l) and NO_3_^−^ in a hydroponic culture. They found that *S. sesban* can grow without an external inorganic N supply by fixing atmospheric N_2_ gas via root nodules. They also found that the addition of external concentrations NH_4_^+^ and NO_3_^−^ alone or mixed at a range of 0, 0.1, 0.2, 0.5, 2, and 5 mM stimulated the growth of seedlings of *S. sesban*. Resulting relative growth rates (RGRs) range from 0.19 (RGRs)/day to 0.21 (RGRs)/day. The authors concluded that these characteristics of *S. sesban* concerning N nutrition make it a very useful plant as N_2_-fixing fallow crop in N-deficient areas. Thus, *S. sesban* has a broad ecological amplitude concerning N nutrition, and the wide geographical distribution of this species in subtropical and tropical areas may in part be due to its adaptability to a variety of environmental conditions, including water regime and nutrient availability. *S. sesban* has great use in tropical and subtropical areas not only as a N_2_-fixing fallow crop in nutrient-deficient areas but also as a recommended species for use in constructed wetland systems for the treatment of NH_4_^+^-rich waters.

#### 3.6.6. Treating Wastewater in Tropical Areas

Dan et al. [107] evaluated the potential of using *S. sesban* as an N_2_-fixing plant in constructed wetland systems. *S. sesban* plants grew well in the vertical flow and horizontal flow systems. The parameters measured such as root elongation rate, shoot elongation rate, leaf production rate, and biomass production were generally high in the two systems. The biomass production for the experimental periods was 20.2 and 17.2 kg/m^2^/year for the vertical flow and horizontal flow systems, respectively. The nitrogen content in *S. sesban* biomass was relatively high in general. They concluded that *S. sesban* can be used to treat high-strength wastewater in tropical areas, while the species grows well and produces a large amount of nitrogen containing biomass which is used as fodder and for soil amendment [107].

#### 3.6.7. Gas Production and Rumen Degradation Characteristics

*S. sesban* leaves have been investigated *in vitro* and in *sacco* in rumen fistulated cows fed on a diet of grass hay ad libitum supplemented with cotton seed cake [72]. The results showed that *S. sesban*' leaves are used for gas production (methane, carbon dioxide) and rumen degradation characteristics, as well as for the growth of *S. sesban* leaves (in heavy clay vertisols with near neutral to alkaline pH (6–8)). They offered advantages over herbaceous species in terms of superior persistence, higher dry matter (DM) yields, better resistance to mismanagement, and a capacity to retain high-quality foliage livestock depending on grazing unimproved native under stress conditions. It also provides fertilizer in the pastures and crop residues and can play a role as protein supplements to poor quality forages [72]. Details of other uses of *S. sesban* discussed in this section are summarized in Table 7.

## 4. Conclusion

*S. sesban* is a leguminous tree native to Chad used to increase crop yields and vegetation in some desert areas. The local population and particularly pastoralists plant it in arid zones to increase the plant cover, to obtain shade for humans and their animals, and to use its wood for construction. The local population also uses *S. sesban* to enrich soil fertility for increasing yields of crops such as rice, maize, and sorghum. The species is also used also as a medicinal plant to treat breast cancer, wounds, and edema. However, some important problems exist which threaten *S. sesban* and other leguminous trees in Chad. Van der Plas and Abdel-Hamid [102] pointed out that around cities such as N'djamena , the high demand for fuelwood threatens the sustainability of supply. They also explained that this demand for fuelwood does not need to be a problem; if supplies are made available, then woodfuel can also be an engine of economic growth, particularly in rural areas.

Land degradation, the decline of soil fertility and carbon stocks, and reduced availability of fuelwood and livestock feed are key problems in Chad as well as throughout Africa. Nwilo et al. [108] noted that vegetation in northern Nigeria including the region of Lake Chad declined by 49.3% between 1984 and 2016. The causes included agricultural activities such as extensive grazing and annual cropping, deforestation, and variations in climate. Excessive exploitation poses risks to the conservation of the whole flora in these and other tropical and subtropical regions [109, 110]. Moreover, effective strategies for biodiversity conservation should focus on regions with rare and endangered species, on locally abundant species that are functionally vital in maintaining the plant community, and on regions with considerable heterogeneity of vegetation [110]. Rukangira [109] noted that policy makers, other stakeholders, and citizens need to support conservation and help increase awareness of the problem. The collection of plant material and documentation, botanical identification, and preparation of herbarium vouchers are tasks that cannot be automated and thus require specialists who are becoming increasingly rare [111, 112]. As reported by Mosier et al. [113], restoring soil fertility on degraded lands to meet food, fuel, and climate security requires perennial cropping systems using perennial vegetation and thus can simultaneously provide additional ecosystem services. These alternative combinations of ecosystem services are climate change mitigation (bioenergy cropping systems), animal protein production (intensive rotational grazing), and biodiversity restoration (conservation plantings).

Finally, this review study demonstrates that *S. sesban* has considerable potential for addressing problems such as land degradation, decline of soil fertility and carbon stocks, and reduced availability of fuelwood and livestock feed as well as for improving human and livestock health and treating diseases. Various phytochemical constituents with essential metabolites and drugs can be extracted from different parts of *S. sesban* including the leaves, bark, stem, flower, roots, and seedpods. *S. sesban* also has important uses in particular niches, such as on saline soils, constructed wetlands, and for phytoextraction of metals. This study contributes to the knowledge base on *S. sesban* and will hopefully help in its protection, use, and value for future generations. Although the plant grows naturally in and around N'djamena , it has become rare and needs to be replanted in order to avoid its complete disappearance in the future.

## Figures and Tables

**Table 1:** *S. sesban* taxonomic classification [15, 20, 21].

*S. sesban* Merr. (L.)	
Kingdom	Plantae
Division	Magnoliophyta or angiosperms dicotyledonous (flowering plants)
Tribe	Robinieae
Class	Magnoliopsida
Order	Fabales
Family	Fabaceae
Subfamily	Papilionaceae
Gender	*Sesbania*
Species	*S. sesban* Merr. (L.)

**Table 2:** Medicinal use, biological activity, plant parts used, dosage regimen, and its corresponding concentrations and active constituents of *S. sesban*.

	Biological activity	Plant part used and optimal solvents	Dosage regimen and corresponding concentrations	Active constituents	References
Medicinal use	Antioxidant activity	Leaves, seeds (ethanolic and methanol extracts)	100 *µ*g/ml after one and half hour 1 mg/ml after one and half hour	Saponins, flavonoids, anthocyanins	[40, 41]
	Antimicrobial activity	Flower petals (methanol and acidified methanol)	1 mg −12.5 mg/ml after 24 hours	Anthocyanins	[41]
	Antimicrobial activity	Bark (ethanol, diethyl ether, chloroform)	250 *μ*g–500 *μ*g: ml after 18 hours 250–8000 *µ*g/ml for the MIC after 12 hours	Carbohydrates, flavonoids, steroids, alkaloids, tannins, saponins	[50]
	Anthelmintic activity	Leaves, seed (aqueous extracts) leaf (hydroethanolic and aqueous extracts)	2.5 mg/ml after 6 hours 5 and 10 mg/mL	Saponins, glycosides saponin, flavonoids, betulinic and ursolic acids	[51–54]
	Antischistosomal effect against the parasite *Schistosoma mansoni* infected the mice	Leaf powder	1000 mg/kg/day for 9 weeks post infection (PI)	Methanol extract	[55]
	*S. sesban* has highly significant antibacterial and antifungal activity	Stem (methanol extracts)	250 *µ*g/ml and 500 *µ*g/ml for bacteria at 37°C after overnight. 100 *µ*g/ml and 500 *µ*g/ml for fungi at 28 ± 2°C after 48 hours	Carbon tetrachloride partitionate, alkaloids, flavonoids, tannins, phytosterols	[42]
	Cytotoxic activity	Bark (ethanol, diethyl ether, chloroform)	320–1280 *µ*g/ml after 24 hours	Carbohydrates, flavonoids, steroids, alkaloids, tannins, saponins	[50]
	Anti-inflammatory activity	Leaves (methanol)	500 mg/kg after 3 hours until 22 days. 250 mg/kg after 3 hours	Saponins	[32, 43]
	Antidiabetic activity	Leaves (aqueous extracts)	250 and 500 mg/kg after day	Triterpenoids, tannins, saponins, glycosides, steroids	[57]
	*S. sesban* roots extract exhibited significant antihyperglycemic activities in streptozotocin STZ-induced diabetic mice	Roots (petroleum ether extract)	200–1000 mg/kg after 2 hours and blood samples were withdrawn until 15 days	Phytosterols, fixed oils, fats, saponins, proteins, gums, mucilage and amino acids)	[58]
	Antinociceptive activity (analgesic)	Wood (petroleum ether, chloroform, ethyl acetate)	50 and 100 mg/kg after 30 minutes	Sterols, triterpenes, flavonoids	[59]
	Control the fertility of female albino rats	Seeds (distilled water)	250 and 400 mg/kg/day for 30 days	—	[61]
	Potent spermicidal activity	Roots (ethylacetate, n-butanol saturated, ethanol, and water)	50 mcg/ml after one hour	Oleanolic acid 3-beta-d-glucuronide	[62]
	Central nervous system stimulant	Bark (aqueous extract)	400 mg/kg after 48 hours	Carbohydrate, alkaloids, phytosterols	[63]
	Molluscicidal activity	Leaves (methanol and aqueous extracts)	LC_0_ = 1.8 ppm to LC_90_ = 31 ppm after 24 hours for aqueous extracts. LC_0_ = 5.11 ppm to LC_90_ = 62.4 ppm after 4 weeks for aqueous extracts	Saponins	[64, 65]
	Antioxidant, antimicrobial, antiviral, anthelmintic, molluscicidal, antifertility, anti-inflammatory, antidiabetic, antihyperlipidemic, anticancer, antianxiety, mosquito repellent properties	Leaves	—	Steroids, triterpenoids, saponins, flavonoids, coumarins, lipids, and other miscellaneous compounds	[48, 49]

**Table 3:** *S. sesban*: phytochemical compounds, parts used for extraction, and their extraction solvent.

Phytochemical compounds	Parts used for extraction	Extraction solvents	References
Sterols, saponins, flavonoids, alkaloids, fats and oil, proteins, sterols, anthraquinone glycosides, gums, and miscellaneous compounds	Leaves	Methanol, chloroform, and petroleum ether 60–80°	[32]

Triterpenoids, carbohydrates, vitamins, amino acids, proteins, tannins, saponins, glycosides, and steroids	Leaves	Aqueous extracts	[57]

Carbohydrates, flavonoids, steroids, alkaloids, tannins, and saponins	Bark	Ethanol ether (diethyl ether) chloroform	[50]

(i) Cyanidin and delphinidin glucosides	(i) Blossoms	Aqueous extracts	[68–70]
(ii) Alpha-ketoglutaric, oxaloacetic, and pyruvic acids	(ii) Dust (pollen) and dust tubes		
(iii) campesterol cholesterol, beta-sitosterol, triterpenoids, proteins, and tannins	(iii) Leaf and pods		
(iv) Glucose, fructose, erythritol, arabinitol, and myo-inositol	(iv) Bark and stem		
(v) Guaiacyl, syringyl, p-hydroxyphenylpropane, and kaempferol	(v) Lignin		

Sterols, triterpenes, and flavonoids	Wood	Petroleum ether and chloroform ethyl acetate	[59]

Carbohydrates, alkaloids, phytosterols, saponins, glycosides, and phenolic compounds	Bark	Petroleum ether, chloroform and aqueous extracts	[63]

Alkaloids, carbohydrates, proteins, phytosterol, phenol, flavonoids, fixed oil, and gum	Stem, roots and leaf	Methanol, ethanol	[42]

Dry matter, crude protein, gas production, ash, neutral detergent fibre, acid detergent fibre, neutral detergent fibre bound nitrogen, soluble proanthocyanidins, quercetin, and saponin	Leaves	—	[71, 72]
Anthocyanins, phenols, and flavonoids	Flower	Methanol and acidified methanol	[41]

Oleanolic acid 3-*β*-D-glucuronide	Root	Ethyl acetate and n-butanol saturated extracts	[62]

Steroids, triterpenoids, saponins, flavonoids, coumarins, lipids, and other miscellaneous compounds	Leaves	—	[48]

**Table 4:** Ethnomedicinal use of *S. sesban*, plant part used, dosage regimen, and mode of preparation.

	Disease treated	Plant part used	Mode of preparation	Dosage regimen	References
Ethnomedicinal use	Breast cancer	Leaves, bark, roots	Maceration of leaves for 48 hours or infusion of root and bark	Drink the maceration morning and evening	Ousman B. M., 2024 (unpublished)
	Edema and swollen glands	Leaves	Leaf powder and oil were applied to the body	Apply to the body until cure	
	The retention of urine of cattle (cows, goats, and buffaloes)	Leaves	Fresh leaves and stems administrated orally	—	[73]
	Pain arising out of pox of cattle	Leaves and stem	The leaves are dried in sunlight and then spread over the bodies		[73]
	Treating mastitis of livestock	Root and bark	The Infusion is topically used	Apply to the body until cure	[75]
	Antimalarial	Leaves and aerial parts	The vapour from boiling leaves is inhaled or a decoction of the parts is drunk	Inhaling vapour two times a day for three days	[76, 77]
	Mosquito repellent	Leaves	Wash the bodies of animals with water leaf extracts	Wash the body until cure	[68]
	Repel tsetse flies	Leaves	Leaf decoction is drenched	Until cure	
	Demulcent, anthelmintic purgative, and anti-inflammatory agent in the treatment of eczema	Leaves	—	—	[48]

**Table 5:** Use of *S. sesban* for soil improvement in agriculture and in cropping systems.

	Detail of use	Part used	References
For soil improvement in agriculture and in cropping systems	*S*. *sesban* induces in symbiosis of the root nodules *Rhizobuim* strains and fixes nitrogen on the soil	Whole tree germinated seedlings	[29, 78–80]
	Cultivated in rotation with cotton to enhance nitrogen fertility and improve soil condition	Whole tree	[7, 81]
	*Rhizobium* make them suitable to be used as biofertilizers in unfavourable environmental conditions for legumes cropping	Root nodules	[92]
	*S. sesban* is planted to increase plant cover in desert areas and areas with little vegetation and to enrich soil fertility for increasing yields of crops in sandy soil and in desert areas	Whole tree	[7, 23, 47, 90], (Ousman B. M., 2024) (unpublished)
	*S. sesban* improves fallow systems and enhances agricultural productivity by increasing the yields of maize and sorghum	Whole tree	[3, 5, 94, 95].
	The foliar biomass production of *S. sesban* allows the production of nutrients nitrogen, phosphorus, calcium, magnesium, and potassium when the species is intercropped with *Phaseolus vulgaris* L., *Sorghum bicolor* L., *Zea mays* L., and *Ipomoea batatas* L	Whole tree bark, stems	[23, 35]
	*S. sesban* accessions are used as green manure crops in short fallow and used as sources of organic matter and nitrogen for improvement of poor, nutrient-deficient soils	Seeds accessions whole tree	[84, 86, 87]
	Intercropping sweet potato with *S. sesban* improves yield of the crop	Whole tree	[88]
	Intercropping of *S. sesban* with rice and annual grasses in semiarid conditions for managing weeds and optimizing the yield of dry-seeded rice	Whole tree bark	[23, 89]
	Improved fallows and as herbaceous cover crops	Whole tree	[4, 5, 5, 85]
	Restore eroded soil by fixing N_2_	Whole tree	[18, 83]
	*S. sesban* grows in the salt-affected soils and limits the effect of salinity	Whole tree	[82, 91]

**Table 6:** Use of *S. sesban* as feed for livestock and as food for humans.

	Detail of use	Part used	References
Feed for livestock and food for humans	Potential of improving traditional sheep husbandry by increasing milk and meat production	Leaves and young twigs	[34, 100]
	*S. sesban* has high feed quality	Whole tree	
	*S. sesban* had the highest dry matter digestibility		[96]
	*S. sesban* is used as feed for livestock (cattle, goats, and sheep) and thus contributes to improve food security, income and livelihoods. Leaves and pods of *S. sesban* are fed by ruminants and considered as a high-protein fodder increasing milk productivity	Whole tree leaves and pods	[29, 34, 99, 101]. (Ousman B. M., 2024) (unpublished)
	*S. sesban* is used for humans' food and their nutrition	Seeds	[97]

**Table 7:** Other uses of *S. sesban.*

	Detail of use	Part used	References
Other uses	*S. sesban* biomass is used as firewood for cooking and heating	Whole tree	[46, 85, 102, 104]
	*S. sesban* is used as shade. Windbreaks, cover crops, ornamental plant, as fish poison and sticks for construction and as hedge	Whole tree	[7, 15, 68] (Ousman B. M., 2024) (unpublished)
	As fibre for ropes and fishing nets, and the seeds produce a gum	Stem and thick branches, bark	[46].
	*S. sesban* may enhance chemically the phytoextraction of heavy metals (cadmium, lead, and zinc) from the soil, when 5 mmol EDTA/kg is applied	Whole tree	[105, 106]
	*S*. *sesban* can tolerate relatively high concentrations of ammoniac ion NH_4_^+^ in a hydroponic culture	Whole tree	[106]
	*S. sesban*, as an N_2_-fixing shrub, is used for treatment of polluted water	Whole tree	[107]
	*S. sesban* leaves are investigated *in vitro* and in *sacco* in rumen fistulated cows fed to produce gas and rumen degradation characteristics, higher dry matter (DM) yields, better resistance to mismanagement, and a capacity to retain high-quality foliage livestock depend on grazing unimproved native under stress conditions	Leaves	[72]

## Data Availability

The data that support the findings of this review study are available from the corresponding author upon reasonable request. All data relating to this species S. sesban generated in this review are included in this manuscript.
